# Recent Advances in Nanoparticle-Mediated Antibacterial Photodynamic Therapy

**DOI:** 10.3390/ijms262210949

**Published:** 2025-11-12

**Authors:** Nivedita, Shashwat Sharma, Dyah Ika Krisnawati, Tsai-Mu Cheng, Tsung-Rong Kuo

**Affiliations:** 1 International Ph.D. Program in Biomedical Engineering, College of Biomedical Engineering, Taipei Medical University, Taipei City 11031, Taiwan; d845111002@tmu.edu.tw; 2 International Ph.D. Program in Medicine, College of Medicine, Taipei Medical University, Taipei City 11031, Taiwan; d142112006@tmu.edu.tw; 3 Department of Nursing, Faculty of Nursing and Midwifery, Universitas Nahdlatul Ulama Surabaya, Surabaya 60237, East Java, Indonesia; dyahika@unusa.ac.id; 4 Graduate Institute for Translational Medicine, College of Medical Science and Technology, Taipei Medical University, Taipei City 11031, Taiwan; 5 Taipei Heart Institute, Taipei Medical University, Taipei City 11031, Taiwan; 6 Cardiovascular Research Center, Taipei Medical University Hospital, Taipei Medical University, Taipei City 11031, Taiwan; 7 Graduate Institute of Nanomedicine and Medical Engineering, College of Biomedical Engineering, Taipei Medical University, Taipei City 11031, Taiwan

**Keywords:** antibacterial photodynamic therapy, reactive oxygen species, upconverting nanoparticles, carbon dots, mesoporous silica nanoparticles, liposomes, metal–organic frameworks, artificial intelligence

## Abstract

The escalating threat of antibiotic resistance has prompted the search for alternative antibacterial therapies. Antibacterial photodynamic therapy (aPDT), which utilizes light-activated photosensitizers to generate reactive oxygen species (ROS), offers a promising, non-invasive approach. The aim of this review is to analyze recent advances in nanoparticle-mediated aPDT and synthesize crucial design principles necessary to overcome the current translational barriers, thereby establishing a roadmap for future clinically applicable antimicrobial treatments. Emerging nanoparticle platforms, including upconverting nanoparticles (UCNPs), carbon dots (CDs), mesoporous silica nanoparticles (MSNs), liposomes, and metal–organic frameworks (MOFs), have demonstrated improved photosensitizer delivery, enhanced ROS generation, biofilm disruption, and targeted bacterial eradication. Synergistic effects are observed when aPDT is integrated with photothermal, chemodynamic, or immunotherapeutic approaches. The review further examines the mechanisms of action, biocompatibility, and antibacterial performance of these nanoparticle systems, particularly against drug-resistant strains and in challenging environments such as chronic wounds. Overall, nanomaterial-mediated aPDT presents a highly promising and versatile solution to antimicrobial resistance. Future perspectives include the integration of artificial intelligence to personalize aPDT by predicting optimal light dosage and nanoplatform design based on patient-specific data, rigorous clinical validation through trials, and the development of safer, more efficient nanoparticle platforms.

## 1. Introduction

Antimicrobial resistance (AMR) is an intrinsic and emerging global health emergency endangering decades of success in modern medicine [1]. AMR results from the emergence of drug resistance mechanisms among microorganisms, bacteria, fungi, viruses, and parasites that render antimicrobial agents ineffective [2,3,4]. The overuse and misuse of antimicrobials in various sectors, such as human medicine, agriculture, and animal husbandry, are central to the emergence and transmission of AMR [5]. The consequences of unbridled AMR are disastrous, with direct causation of 1.27 million deaths globally in 2019 and a contributory role in nearly 5 million deaths in the same year [6]. Predictions suggest that if left unchecked, AMR will cause as many as 10 million deaths each year by the year 2050, costing an enormous USD 100 trillion economic price tag. At the same time, microbial infections remain a pervasive and significant global human health threat, demonstrating ubiquitous occurrence within community and healthcare environments.

Considering the interplay between rising AMR and chronic microbial infection load, an interdisciplinary approach encompassing antimicrobial stewardship, infection control, and development of novel therapeutic strategies is urgently required [7,8]. Understanding the mechanisms of action of reactive oxygen species (ROS) and exploring novel ROS-mediated therapies, like photodynamic therapy, is valuable in tackling these problems [9]. It was discovered a few decades ago that PDT leverages agents called photosensitizers (PSs) [9,10,11,12]. These photosensitizers, upon exposure to light of a specific wavelength, form reactive oxygen species (ROS) [13]. Such species are extremely reactive molecules that can effectively destroy microorganisms present in the infection site by causing oxidative damage [14]. In comparison to the traditional antibiotic therapies, PDT possesses several important advantages, including increased biological compliance and safety with a potential for cyclic administration under specific clinical conditions [15,16]. Notably, PDT has been found to be very effective against many antibiotic-resistant bacteria, without simultaneously allowing the emergence of new resistance mechanisms, a significant advantage in the face of increasing antibiotic resistance [17]. Consequently, this therapy has emerged as a promising strategy against the treatment of many infections.

In recent years, the integration of nanotechnology with PDT has revolutionized its therapeutic potential. Nanoscale photosensitizer carriers such as metallic, polymeric, and semiconductor nanoparticles enable improved solubility, targeted delivery, and controlled release of photosensitizers while facilitating deeper tissue penetration and enhanced ROS generation efficiency [18,19,20,21]. Moreover, nanostructured platforms offer multifunctionality by combining photothermal and photodynamic effects, optimizing light absorption, and enabling image-guided antimicrobial therapy. These nanoscale enhancements have significantly improved PDT’s precision, bioavailability, and overall antibacterial efficacy, marking the emergence of nanophotodynamic therapy (nano-PDT) as a leading frontier in antimicrobial innovation [18,19,20,21,22,23,24,25]. Nanomaterial-based PDT possesses several important characteristics, like controlled-release function, high water solubility, good biocompatibility, and the ability to generate massive amounts of ROS [26,27,28]. Therefore, nanomaterial-based PDT is a promising field for antibacterial therapy with the potential to overcome the limitations of current PDT procedures and provide more effective and targeted therapy for bacterial infections.

## 2. Mechanism of Photodynamic Therapy

Photodynamic therapy is an advanced antibacterial therapy channeling upon the local or systemic administration of the photosensitizers (PSs) [29]. Following the exposure to light of a particular wavelength, the photosensitiser undergoes a transformation into different energy states, resulting in ROS formation [14]. The ground-state PSs capture that light energy and achieve excitation to form a highly energetic singlet state (1s*). These singlet-state PSs then undergo intersystem crossing to transition to a triplet state (3s*) and emit energy [30]. Along this process, triplet-state PSs encounter the molecular oxygen available from the immediate surrounding environment to cause the development of ROS [22]. The generated ROS, like singlet oxygen (^1^O_2_), superoxide radical (O_2_^−^), hydroxyl radical (OH·), and hydrogen peroxide (H_2_O_2_), then yield their antibacterial actions by oxidizing vital bacterial structures [31], e.g., DNA, lipids, proteins, and enzymes, which leads to destruction and breakdown of bacterial cell structure and activities (Table 1). However, conventional drug-based PSs possess a few inherent demerits that might limit their clinical application. These limitations include low water solubility, which may hinder their bioavailability and distribution in the body, poor drug release control, leading to erratic therapeutic outcomes, and low selectivity, potentially causing off-target activities on normal tissues [32]. Some drug-based PSs also have the potential to cause unforeseen side effects and damage to normal tissues [33]. In spite of research attempts to develop novel PSs through organic synthesis, the complexity of the process raises concerns about the cost and time required in this process [29]. In order to avoid the limitations of traditional drug-based PSs, most research has been directed towards developing nanoparticles. These nanoparticles are of great potential since they can function as PSs or as carriers of traditional PSs, which enhances their delivery and efficacy. Nanomaterial-based PDT possesses several important characteristics like controlled-release function, high water solubility, good biocompatibility, and the ability to generate massive amounts of ROS [26,27,28]. Therefore, nanomaterial-based PDT is a promising field for antibacterial therapy with the potential to overcome the limitations of current PDT procedures and provide more effective and targeted therapy for bacterial infections.

**Figure 1:**
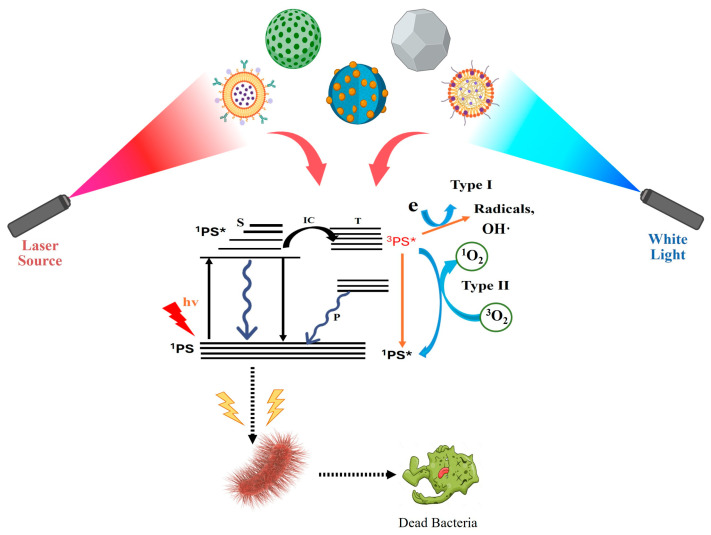
Schematic representation of nanoscale photosensitizer-based antibacterial photodynamic therapy (aPDT) under laser and white-light irradiation. Nanostructured photosensitizers (liposomal, polymeric, and core–shell types) generate reactive oxygen species (ROS) via Type I (radical-mediated) and Type II (singlet oxygen-mediated) pathways. The excited photosensitizer transitions to its triplet state (^3^PS*), producing ROS that damage bacterial membranes and cellular components. The nanoscale design enhances light absorption, ROS yield, and bacterial interaction, resulting in improved antibacterial photodynamic efficacy.

Reactive oxygen species (ROS) are central to the antibacterial mechanism of photodynamic therapy (PDT), inducing oxidative damage to bacterial biomolecules. Upon light activation, photosensitizers (PSs) are excited, promoting electrons from the valence to the conduction band and creating electron–hole pairs [9,10]. These charge carriers undergo redox reactions with surrounding oxygen and water molecules, generating ROS via two primary pathways: Type I, involving electron or hydrogen transfer to yield O_2_^−^, OH·, and H_2_O_2_, and Type II, in which the excited PS transfers energy directly to molecular oxygen to form singlet oxygen (^1^O_2_) [11]. While Type II is oxygen-dependent, Type I can proceed effectively even under hypoxic conditions (Figure 1).

The principal ROS superoxide anion (O_2_^−^), hydrogen peroxide (H_2_O_2_), singlet oxygen (^1^O_2_), and hydroxyl radical (OH·) exert complementary oxidative effects (Table 1). In brief, O_2_^−^ generated by single-electron reduction of O_2_ may further participate in Haber–Weiss or Fenton reactions to form OH· [9,10]. ^1^O_2_ arises from the energy transfer between excited PSs and molecular oxygen, oxidizing nucleic acids, proteins, and lipids [15]. OH·, produced through H_2_O_2_ reduction or surface oxidation reactions [17], is highly reactive and causes localized damage due to its short lifetime [14,16]. H_2_O_2_, formed via O_2_^−^ dismutation or stepwise reduction of O_2_, is relatively stable, able to diffuse through membranes, and interferes with iron–sulfur enzymes [13]; it can also act as a precursor for OH· formation [17]. Collectively, these ROS mediate oxidative stress and membrane disruption, leading to bacterial cell death.

## 3. Novel Nanoparticle Materials

### 3.1. Upconverting Nanoparticles

**Figure 2:**
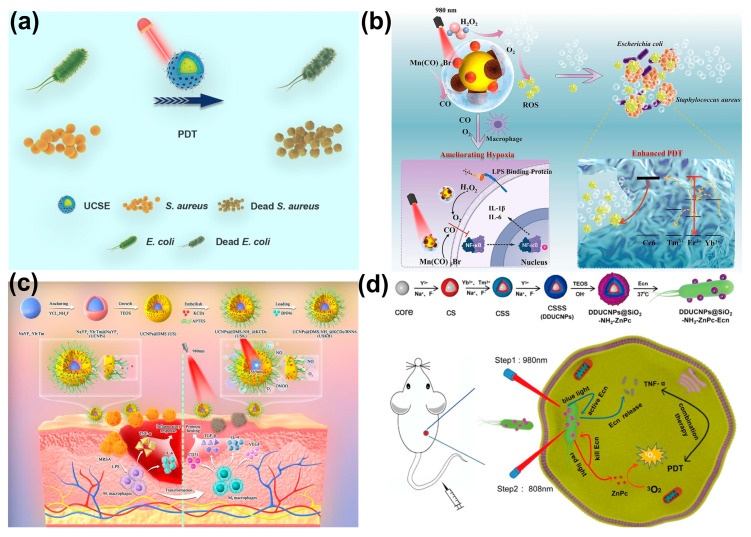
(**a**) Antibacterial mechanism using UCSEs for PDT. (**b**) Antibacterial and anti-inflammatory mechanism of UCM@Si. (**c**) Synthetic route and proposed mechanism of USKB against MRSA biofilm-infected diabetic wounds. (**d**) Schematic diagram of near-infrared-triggered programmed photodynamic and immune combination therapy. Reproduced from open-access references [36,37,39,40].

Upconversion nanoparticles (UCNPs) have emerged as a powerful tool in photodynamic therapy (PDT) due to their unique ability to convert near-infrared (NIR) light into shorter wavelengths, enabling deeper tissue penetration and minimizing damage to superficial tissues. This upconversion process occurs when the UCNP crystal absorbs multiple low-energy NIR photons and combines their energy to emit a single photon at a higher (visible) wavelength, which then activates the photosensitizer [34]. This property has been used to develop innovative nanoplatforms for combating bacterial infections, addressing limitations associated with conventional PDT [35]. Several studies highlight the efficacy of UCNP-based PDT in eradicating bacterial infections. Jing Luo et al. developed UCSE, a system utilizing UCNPs loaded with erythrosine, as shown in Figure 2a, demonstrating significant antibacterial activity against *Staphylococcus aureus* (*S. aureus*) and *Escherichia coli* (*E. coli*) through ROS generation and cell membrane disruption [36]. Wei Liu and colleagues created UCM@Si, a nanocomposite that combines UCNPs with Chlorin e6 (Ce6) and Mn(CO)_5_Br, achieving simultaneous bacterial killing and inflammation control [37]. As shown in Figure 2b, the Mn^2+^ component mitigates hypoxia, enhancing aPDT efficacy, while CO modulates macrophage polarization, facilitating wound healing [38]. Zekun Wang et al. introduced USKB, a nanosystem generating highly lethal peroxynitrite (ONOO^−^) through the synergistic action of kanamycin-derived carbon-nanodots (KCDs) and a nitric oxide donor (BNN6), effectively combating diabetic wound infections as depicted in Figure 2c [39]. These studies collectively underscore the potential of UCNP-mediated aPDT in overcoming antibiotic resistance and promoting wound healing. Beyond antibacterial applications, UCNPs have been integrated with other therapeutic modalities to enhance treatment efficacy. Feiyan Chen and colleagues designed a nanoplatform combining dual-emission UCNPs (DDUCNPs) with modified bacteria, enabling dual-wavelength activation for tumor therapy [40]. This system triggers TNF-α production for tumor ablation and generates singlet oxygen for bacterial killing and enhanced PDT. The utilization of genetically modified bacteria, as demonstrated by the development of upconverting dual-photosensitizer-expressing bacteria (UDPB), further expands the therapeutic potential. Figure 2d shows UDPB expressing both photothermal melanin and phototoxic KillerRed, allowing for combined photothermal therapy (PTT) and PDT under NIR irradiation, demonstrating strong antitumor responses and accelerated wound healing [41,42,43,44].

### 3.2. Carbon Dots (CDs)

**Figure 3:**
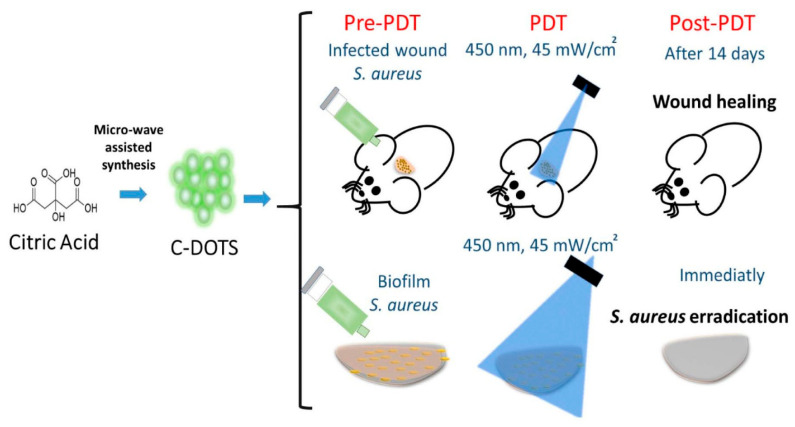
Brief description of the procedures carried out in this study. In vivo and in vitro antibacterial photodynamic therapy (aPDT) studies, where aPDT mediated by C-DOTS and blue LED light against *S. aureus* were evaluated. Reproduced from an open-access reference [47].

The upsurge issue of antimicrobial resistance requires an investigation of innovative antibacterial methods, with photodynamic therapy (PDT) emerging as a viable strategy that employs photosensitizers to produce reactive oxygen species (ROS) for microbial eradication. In this context, carbon dots (CDs), zero-dimensional nanoparticles recognized for their distinctive optical properties and biocompatibility, have attracted considerable interest, showing promise as both independent photosensitizers and synergistic enhancers in photodynamic therapy (PDT) [45]. Their sp^2^ carbon lattice and abundant surface functional groups allow them to act as potent intrinsic photosensitizers, showing promise as both independent therapeutic agents and synergistic enhancers in photodynamic therapy PDT. Their extensive surface area and quantum yield promote effective reactive oxygen species (ROS) generation, establishing them as formidable antibacterial agents, while their adjustable surfaces allow for tailored distribution and enhanced engagement with bacterial cells. Consequently, carbon dots (CDs) are undergoing rigorous examination for their potential to combat bacterial infections, especially those resistant to traditional antibiotics, thereby emphasizing their increasing significance in advanced antimicrobial research [46]. This is exemplified by the study conducted by María Paulina Romero et al., as represented in Figure 3, which demonstrated the effective application of economical, stable carbon dots synthesized from citric acid for potent in vitro and in vivo antimicrobial photodynamic therapy against (*S. aureus*) [47].

Merat Karimi and colleagues synthesized chromium-doped alumina-carbon quantum dot nanoparticles (Al_2_O_3_:Cr/Cdot NPs) that exhibited enhanced reactive oxygen species (ROS) production, leading to pronounced antibacterial efficacy against drug-resistant bacteria and substantial biofilm reduction [23]. This effect is likely attributable to a synergy of alumina’s membrane disruption and ROS generation, coupled with augmented anticancer activity under UVA exposure, indicating their potential as multifunctional agents in nanomedicine for addressing drug-resistant infections and advancing photodynamic cancer therapy.

### 3.3. Mesoporous Silica Nanoparticles

Mesoporous silica nanoparticles (MSNs) are a class of nanocarriers widely explored in biomedicine due to their unique physicochemical properties. Their rigid SiO_2_ framework, characterized by high surface area and tunable pore size, functions primarily to provide highly efficient encapsulation, protection, and controlled release of therapeutic agents [48]. In antibacterial photodynamic therapy (aPDT), MSNs serve as versatile platforms that enhance the solubility, stability, and targeted delivery of photosensitizers, while promoting reactive oxygen species (ROS) generation and effective biofilm disruption [49]. Their easily modifiable surfaces allow for functionalization with targeting ligands or stimuli-responsive gates, further improving bacterial adherence and therapeutic precision [50]. Collectively, these properties position MSNs as highly promising nanoplatforms for enhancing the efficacy and safety of aPDT in the treatment of drug-resistant infections.

**Figure 4:**
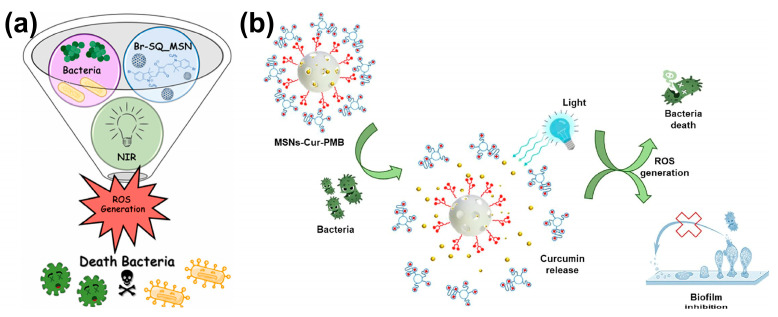
(**a**) Mechanism. Reproduced with authorization from an open-access reference [52]. (**b**) Mechanism of MSNs–Cur–PMB. Reproduced with authorization from a reference [53].

The promising potential of mesoporous silica nanoparticles (MSNs) in improving antimicrobial photodynamic treatment (aPDT) is shown by recent studies [51]. Effective transporters for photosensitizers, these nanoparticles enhance their delivery and stability, therefore facilitating efficient bacterial eradication. Degnet Melese Dereje and colleagues posited that encapsulating the hydrophobic squaraine dye (Br-SQ) within mesoporous silica nanoparticles (MSNs) would enhance its solubility, thereby reducing aggregation and augmenting its efficacy as an antibacterial photodynamic therapy (aPDT) agent [52]. The successful manufacturing and characterization of Br-SQ-loaded MSNs demonstrated effective dye integration and the preservation of suitable nanoparticle properties for biological applications. As shown in Figure 4a, the nanoformulation exhibited significant antibacterial efficacy against both Gram-positive *S. aureus* and Gram-negative *E. coli* at nanomolar concentrations, despite a reduction in ROS production compared to free Br-SQ. The improved efficacy at lower concentrations, attributed to superior dye delivery and diminished aggregation, suggests that MSNs serve as effective nanocarriers for hydrophobic photosensitizers, offering a viable approach for creating antibiotic-free nanoformulations to combat bacterial infections and mitigate antimicrobial resistance. For photodynamic treatment (aPDT), Serena Medaglia and colleagues have produced a new antimicrobial nanodevice, MSNs–Cur–PMB, consisting of mesoporous silica nanoparticles loaded with curcumin (Cur) and gated with polymyxin B (PMB) [53]. Combining the antibacterial action of PMB to fight germs with the aPDT characteristics of Cur, activated by blue LED light (470 nm), the nanodevice creates synergy. Compared to free Cur or PMB at equal dosages, testing against *E. coli*, *Pseudomonas aeruginosa*, and *Staphylococcus epidermidis* revealed much improved antibacterial efficacy. In particular, 1 µg/mL was efficient against *P. aeruginosa* and *S. epidermidis*; 0.1 µg/mL of MSNs–Cur–PMB plus light irradiation eradicated roughly 10^5^ CFU/mL of E. coli. Moreover, the nanodevice showed strong antibiofilm action, thereby preventing biofilm development by 99% for *E. coli* (0.1 mg/mL) and almost totally for *S. epidermidis* and *P. aeruginosa* (1 mg/mL). The synergistic mix of Cur and PMB, the protective impact of encapsulating inside the nanoparticles, and the regulated release of the antimicrobial agents upon bacterial engagement explain this increased activity. The results imply that MSNs–Cur–PMB is a promising antibacterial and antibiofilm agent, as represented in Figure 4b. The study also notes that although PMB is usually more effective against Gram-negative bacteria due to its interaction with LPS [54], the observed activity against the Gram-positive *S. epidermidis* may be due to interactions with teichoic acids [55,56,57]. Although its inherent limitations, such as poor solubility and photodegradation, are admitted, the increased activity of Cur upon irradiation is ascribed to the formation of singlet oxygen [58]. While the gating mechanism guarantees regulated release [59,60,61], the encapsulation of Cur and PMB inside the mesoporous silica nanoparticles is thought to protect these molecules and improve their activity [62,63,64].

**Figure 5:**
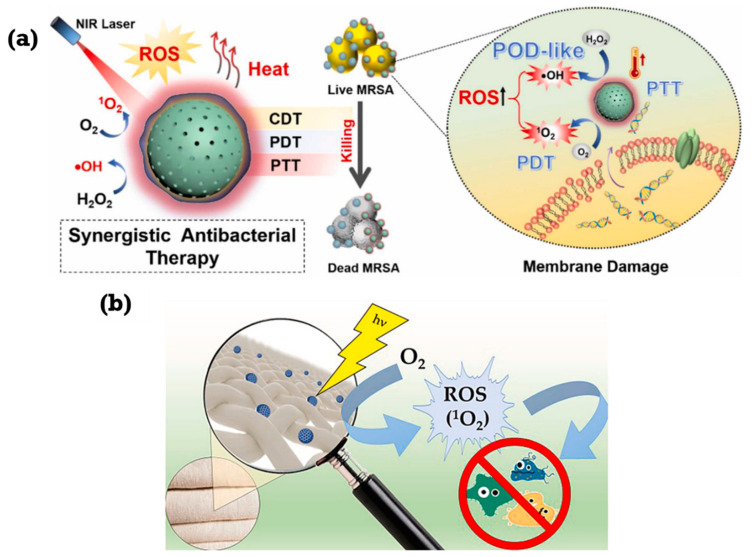
(**a**) Schematic illustration of the preparation process of MSN-ICG@PB and (**b**) the triple-mode synergistic antibacterial mechanisms. Reproduced with authorization from references [65,66].

A novel nanoplatform, MSN-ICG@PB, was synthesized for synergistic photothermal/photodynamic/chemodynamic therapy (PTT/PDT/CDT) against methicillin-resistant *S. aureus* (MRSA), demonstrating high MRSA killing efficiency (>98%) through combined hyperthermia, singlet oxygen generation, and hydroxyl radical production upon NIR laser stimulation and H_2_O_2_ addition, as presented in Figure 5a. This system, featuring indocyanine green (ICG) encapsulated within mesoporous silica nanoparticles (MSNs) and coated with Prussian blue (PB), exhibited low cytotoxicity and high biocompatibility, highlighting its potential for combating drug-resistant bacteria [65]. Additionally, Figure 5b shows research on antimicrobial textiles has explored the integration of dye-loaded MSNs into fabrics, achieving significant antibacterial activity against *S. aureus* and *E. coli* through photodynamic inactivation, with controlled dye release and enhanced stability. These studies underscore the versatility of MSNs in developing advanced antimicrobial strategies, ranging from targeted nanotherapy to functional textiles, by leveraging their unique properties for controlled drug delivery and enhanced therapeutic efficacy [66]. Xiaojiang Huang and colleagues effectively synthesized a novel drug delivery system (MSN-PEG-Hypericin) by combining mesoporous silica nanoparticles, PEG, and hypericin, a photosensitizer [67]. While showing acceptable biocompatibility (low cytotoxicity and hemolysis), characterization validated successful synthesis, and in vitro studies revealed that MSN-PEG-Hypericin improved hypericin’s reactive oxygen species (ROS) production, vital for photodynamic treatment (PDT). With *S. aureus* more susceptible, probably due to structural variations in their cell walls leading to differing hypericin absorption, antimicrobial experiments against *S. aureus* and *E. coli* revealed MSN-PEG-Hypericin had greater photodynamic antibacterial activity than free hypericin. Increased calcium ion concentration and nucleic acid content in treated bacteria indicate that ROS-mediated bacterial cell destruction causes leakage of cellular contents. Improved hypericin solubility and dispersion in MSN-PEG-Hypericin compared to the free form explain the greater ROS generation by it. Especially in the presence of esterase, the continuous release of hypericin from the delivery system supports its possible for regulated PDT.

### 3.4. Polymer-Based Nanoparticles

Polymer-based nanoparticles represent a highly adaptable class of drug delivery systems. Their flexible, organic polymeric backbone allows for facile chemical modification, granting precise control over physicochemical properties (size, charge, degradation rate) to achieve sustained release, improved stability, and targeted binding [68]. Their excellent biocompatibility and ability to encapsulate both hydrophilic and hydrophobic molecules make them well-suited for biomedical applications. In the context of antibacterial photodynamic therapy (aPDT), these nanoparticles can significantly enhance the solubility, stability, and bioavailability of photosensitizers. Moreover, they offer sustained and controlled release, improved cellular uptake, and targeted delivery, crucial for maximizing reactive oxygen species (ROS) generation while minimizing off-target effects [69,70]. These advantages make polymeric nanoparticles highly promising platforms for improving the efficacy of aPDT in the treatment of bacterial infections.

**Figure 6:**
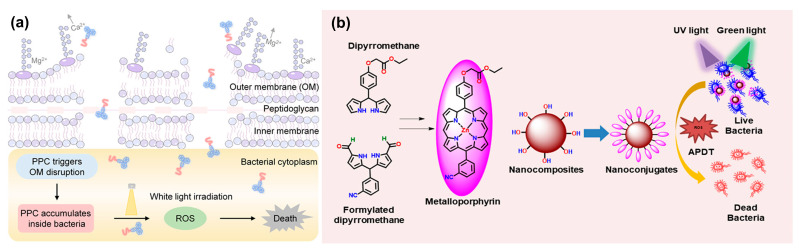
(**a**) Mechanism of peptide–photosensitizer conjugate. (**b**) Mechanism of ZnOAL@EP and ZnOKL@EP. Reproduces with authorization from the reference [71,82].

Developed to improve photodynamic treatment (PDT) against Gram-negative bacteria was an amphipathic peptide–photosensitizer conjugate (PPC) [71]. PPC binds to and disturbs the outer membrane (OM) of Gram-negative bacteria, including *P. aeruginosa* and *E. coli*, by combining a hydrophilic cationic peptide with a hydrophobic photosensitiser (Figure 6a). This disturbance lets the photosensitizer pass through the bacteria, where it produces singlet oxygen upon light irradiation, therefore killing the bacteria. Significantly surpassing the photosensitizer alone, in vitro experiments showed PPC achieved near-complete eradication of Gram-negative bacteria (99.9% for *P. aeruginosa* and 99.9999% for *E. coli*) at 32 μM with light exposure [72]. In mouse models, in vivo PPC-mediated PDT effectively cured bacterial keratitis brought on by *P. aeruginosa* and full-thickness skin infections, therefore encouraging wound healing and eliminating corneal infections [73,74,75]. Mechanistic investigations showed that PPC binds to both lipopolysaccharide (LPS) and phospholipids to disturb the OM and therefore enable bacterial penetration and subsequent PDT efficacy [76]. Against Gram-negative microorganisms, this dual-action mechanism, membrane disturbance, and PDT, presents a promising broad-spectrum antibacterial strategy addressing the challenge of their OM impermeability. The peptide’s capacity to disturb the OM, therefore enabling higher penetration and singlet oxygen formation inside the bacterium, explained the improved PDT effect of PPC over the photosensitizer alone. Further verifying the OM’s function in reducing photosensitizer potency were studies using bacterial spheroplasts (lacking an OM) [77,78]. It was demonstrated that OM disturbance depends critically on the interaction of PPC with LPS and phospholipids, which increases bacterial sensitivity to PDT [79,80,81]. In vivo investigations confirmed the in vitro results and showed PPC’s therapeutic ability for Gram-negative bacterial infections.

For increased antimicrobial photodynamic treatment (aPDT), novel hydrophilic nanoconjugates (ZnOAL@EP and ZnOKL@EP) were produced by conjugating a metalloporphyrin (EP) with lignin-based zinc oxide nanocomposites (ZnOAL and ZnOKL) [82]. Based on their stability and electronic characteristics, computational investigations (TD-DFT) found EP to be a good photosensitizer (Figure 6b). Key in aPDT, the resultant nanoconjugates, characterized by a particle size of 5–20 nm and a porphyrin loading of ~30%, showed improved singlet oxygen production upon dual light (UV + green) exposure. These nanoconjugates were also pH-responsive, releasing ~94% of the porphyrin at pH 5.5 compared to ~25% at pH 7.4, which qualifies them for focused drug delivery in acidic bacterial surroundings. Under dual light irradiation, in vitro tests against *E. coli* revealed a 4–7-fold increase in aPDT efficiency with the nanoconjugates over the native probes, obtaining IC_50_ values of 20.08 and 10.39 μg/mL for ZnOAL@EP and ZnOKL@EP, respectively. The higher singlet oxygen creation and ROS formation by the nanoconjugates upon dual light activation are responsible for this enhanced antibacterial action; bacterial cell membrane disruption and cytoplasmic leakage result from both processes [83]. These results imply that pH-responsive drug delivery systems and topical infection antibacterial treatments based on metalloporphyrin have great potential. Computational studies demonstrating EP’s enhanced stability and charge induction capabilities [84], successful conjugation and characterization of the nanoconjugates [85], and the demonstration of enhanced singlet oxygen generation and ROS production leading to improved antibacterial activity [86,87,88,89,90,91].

**Figure 7:**
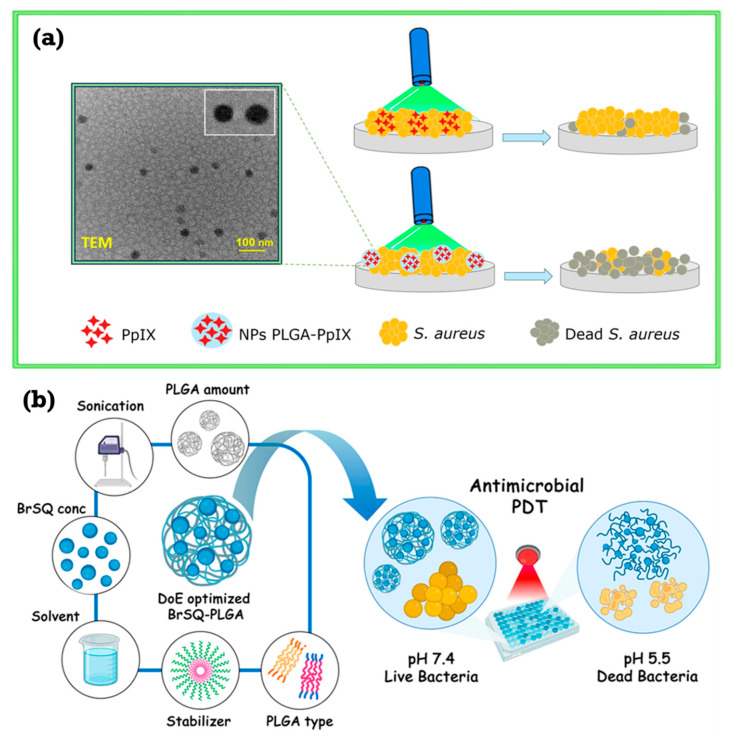
(**a**) Mechanism aPDT against *S. aureus* using polymeric nanoparticles. (**b**) Mechanism of BrSQ-PLGA NPs. Reproduced from open-access references [92,107].

Antimicrobial photodynamic therapy (aPDT) against *S. aureus* was achieved through the development of polymeric nanoparticles that encapsulate the endogenous photosensitizer protoporphyrin IX (PpIX) [92]. While concurrently lowering photobleaching and cytotoxicity in mammalian cell cultures, encapsulation of PpIX into PLGA nanoparticles conserved its photodynamic antibacterial activity relative to free PpIX (Figure 7a). The capacity of the nanoparticles to raise PpIX’s water solubility, photostability, and offer a continuous release of the photosensitizer helps to explain this improved performance. The mechanism of action involves PpIX releasing from the nanoparticles, followed by light irradiation, which produces reactive oxygen species (ROS), therefore killing bacteria. While decreasing dark toxicity, this nanoencapsulation technique presents a viable means to overcome constraints related to hydrophobic photosensitizers like PpIX, such as poor solubility and photobleaching. Studies demonstrating higher ROS generation and lower photobleaching of PpIX compared to other photosensitizers like ICG [25,93,94,95,96,97], the successful encapsulation and controlled release of PpIX from PLGA nanoparticles [98,99,100,101], and the shown antimicrobial activity and reduced cytotoxicity of the PpIX-loaded nanoparticles support these conclusions [102,103,104,105,106]. While the persistent release stops recolonization, the first burst release of PpIX allows for fast diffusion and pathogen photoinactivation.

The potential of brominated squaraine (BrSQ) integrated into poly lactic-co-glycolic acid (PLGA) nanoparticles (NPs) as a photosensitizer for antimicrobial photodynamic therapy (aPDT) was investigated in the study by Degnet Melese Dereje and colleagues [107]. By using single emulsion and nanoprecipitation techniques, researchers adjusted BrSQ-PLGA NPs using a design of experiments (DoE) methodology to assess the impact of different parameters on particle size, zeta potential, yield, and encapsulation efficiency (Figure 7b). For BrSQ encapsulation, the single emulsion technique proved better. Characterization of the nanoparticles verified effective BrSQ integration without changing particle size or form. Though somewhat less than in free BrSQ, the NPs maintained in vitro reactive oxygen species (ROS) production. Under several settings, antimicrobial efficacy against *S. aureus* was assessed; the most efficient aPDT was found at higher dye concentrations, increased light irradiation and fluence, and an acidic pH of 5.5. This higher activity at lower pH is most likely the result of greater light penetration through a swollen, porous PLGA matrix. The study notes the need for more optimization to reach better bacterial inactivation rates for future in vivo uses, even while showing encouraging outcomes. This study overcomes BrSQ’s solubility and aggregation problems by encapsulating it in PLGA NPs, building on past studies showing strong ROS generation [108,109]. DoE enabled effective NP synthesis to be optimized; the choice of PLGA was based on its biodegradability, biocompatibility, and FDA/EMA approval [110,111,112,113,114]. Additionally, taken into account in the study were the effects of the PLGA lactide/glycolide ratio on drug release and the advantages of Pluronic F-127 as a stabilizer [21,115]. The reported pH-dependent increase in aPDT activity corresponds with the acidic environment of bacterial infections and the known breakdown of PLGA under acidic conditions [116,117].

Ya-li Xiang and colleagues synthesized poly-l-lysine (PLL) modified metal–organic framework (MOF) nanoparticles ZIF/PLL-CIP/CUR for synergistic chemo-photodynamic therapy against drug-resistant bacterial infections, particularly methicillin-resistant *S. aureus* (MRSA) [118]. Common antibiotics ciprofloxacin (CIP) and curcumin (CUR), a photosensitizer, are included in the nanoparticles. Under blue light irradiation, CUR generates reactive oxygen species (ROS), whereas CIP is released in reaction to the ROS generated by the bacterium, therefore producing a synergistic effect. Showing good biosafety at physiological pH and targeted release in the bacterial microenvironment, the nanoparticles displayed pH-sensitive and ROS-responsive drug release. Effective MRSA killing and biofilm breakdown via the combination therapy were shown in vitro investigations. The nanoparticles showed great biosafety in vivo and a 98.81% healing rate in a mouse model of MRSA infection, underlining their possible resistance against drug-resistant bacterial infections. This research uses the MOF-based delivery system for improved medication administration and synergistic therapy, building on the use of CUR as a photosensitizer for ROS production. Furthermore, examined in the paper are the mechanism of ROS generation by CUR and the synergistic effect of combination chemo-photodynamic therapy in planktonic and biofilm MRSA. The in vivo studies indicate even more the biocompatibility and efficiency of the nanoparticles in treating MRSA infections.

### 3.5. Liposomes

Liposomes are nanoscale vesicles composed of one or more phospholipid bilayers. Their structural mimicry of biological membranes and intrinsic biocompatibility are key to their function, allowing them to effectively encapsulate both hydrophilic and hydrophobic photosensitizers, enhance selective accumulation at infection sites, and minimize systemic toxicity [119]. Liposomal encapsulation can enhance the selective accumulation of photosensitizers at infection sites, protect surrounding healthy tissue, and reduce systemic toxicity [120]. These advantages make liposomes highly promising nanocarriers for improving aPDT outcomes, particularly in targeting multidrug-resistant bacterial infections.

**Figure 8:**
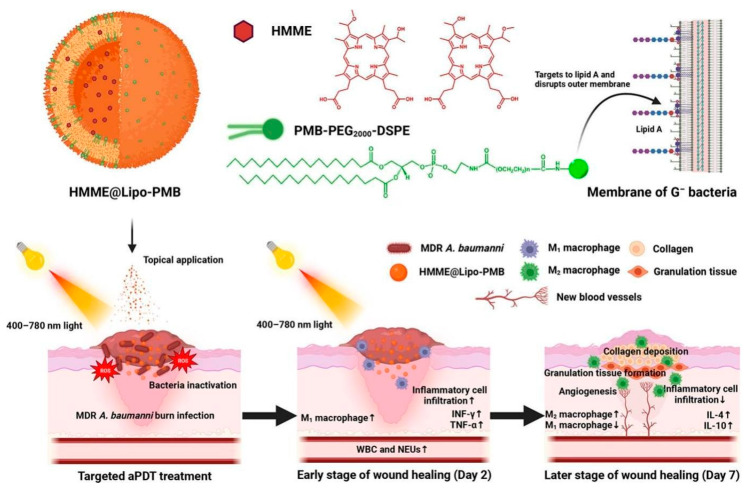
Mechanism of HMME@Lipo-PMB to improve antimicrobial photodynamic treatment. Reproduced with permission from the reference [121].

Zixin Cui and colleagues developed HMME@Lipo-PMB, a new polymyxin B (PMB)-targeted liposomal photosensitizer for improved antimicrobial photodynamic treatment (aPDT) against burn infections caused by multidrug-resistant (MDR) *Acinetobacter baumannii* [121]. Increased intracellular ROS generation and bacterial death both in vitro and in vivo resulted from HMME@Lipo-PMB showing notably better binding to *A. baumannii* than HMME or HMME@Lipo alone (Figure 8). At lower doses than HMME or HMME@Lipo, in vitro HMME@Lipo-PMB attained full bacterial inactivation. In vivo HMME@Lipo-PMB aPDT dramatically enhanced survival rates and completely cleared MDR *A. baumannii* burn infections in mice [122]. Moreover, by adjusting macrophage polarization (M_1_ to M_2_ transition) and modulating the inflammatory response, HMME@Lipo-PMB aPDT stimulated acute inflammation in early stages and therefore reduced chronic inflammation later. Emphasizing its promise as a viable treatment approach for MDR *A. baumannii* burn infections, this focused aPDT also promoted granulation tissue development, angiogenesis, and collagen regeneration. Previous studies showing the efficacy of aPDT against MDR *A. baumannii* employing different photosensitizers, the benefits of liposomal encapsulation for photosensitizers, and the use of PMB for targeted delivery of antimicrobials support these results [123]. Using bioluminescent *A. baumannii* strains, the study additionally expands on current in vivo burn infection models and fits findings on the function of macrophage polarization in wound healing. Enhanced binding of HMME@Lipo-PMB to bacterial cells resulting from PMB drives greater ROS formation upon light activation, disturbs bacterial cell membranes, and finally results in bacterial death [124]. Furthermore, the targeted aPDT controls the immune response, therefore encouraging a change from pro-inflammatory M_1_ macrophages to anti-inflammatory M_2_ macrophages, which are crucial for tissue regeneration and wound healing.

Swagatika Panda and colleagues developed and characterized nanoliposomal improved Toluidine Blue O (NLITBO) as a possible antibacterial agent against *Streptococcus mutans*, a main cause of dental caries [125]. The thin-film hydration technique produced improved Toluidine Blue O (ITBO), which was synthesized and encapsulated in nanoliposomes (NL). Characterizing the resultant NLITBO, it showed a polydispersity index of 0.57, a vesicle size of 123.52 nm, a zeta potential of −39.54 mV, 9.3% drug loading, 84.4% loading efficiency, and a 73.5% yield. 79.81% ITBO from NLITBO was shown to be continuously released in vitro drug release experiments throughout 24 h. Assessed by zone of inhibition (ZOI) and minimum inhibitory concentration (MIC), NLITBO showed substantial antibacterial efficacy, equivalent to 2% chlorhexidine gluconate, even in the absence of light activation. Determined to be 0.6 μg/mL, the MIC of NLITBO as a photosensitizer with red light (650 nm, 0.1 W/cm^2^, 9–9.1 J/cm^2^, 90 s) was calculated. These results imply that NLITBO, with its improved stability, cellular absorption, and possible synergistic effects from the nanoliposome carrier, presents promise as a successful substitute for addressing *Streptococcus mutans* infection in dental healthcare. The impressively low MIC of 0.6 μg/mL for NLITBO represents a significant increase in photodynamic efficacy compared to the free, unencapsulated ITBO dye (MIC approx 2.5 μg/mL), clearly demonstrating the enhanced cellular absorption and delivery benefit provided by the nanoliposomal carrier. Supporting earlier studies on the advantages of nanoliposomal encapsulation for antimicrobial photodynamic therapy and the use of TBO in combination with other agents to combat bacterial infections, the study emphasizes the need for ITBO purity and delivery system in enhancing its efficacy as a photosensitizer. Effective clinical application depends on the continuous release of ITBO from NLITBO, which therefore points to the possibility for extended therapeutic benefits. By optimizing light parameters and employing clinical strains of *S. mutans*, more relevant to clinical settings, the study also addressed the variability in TBO concentration found in earlier studies.

## 4. Combination Therapies

**Figure 9:**
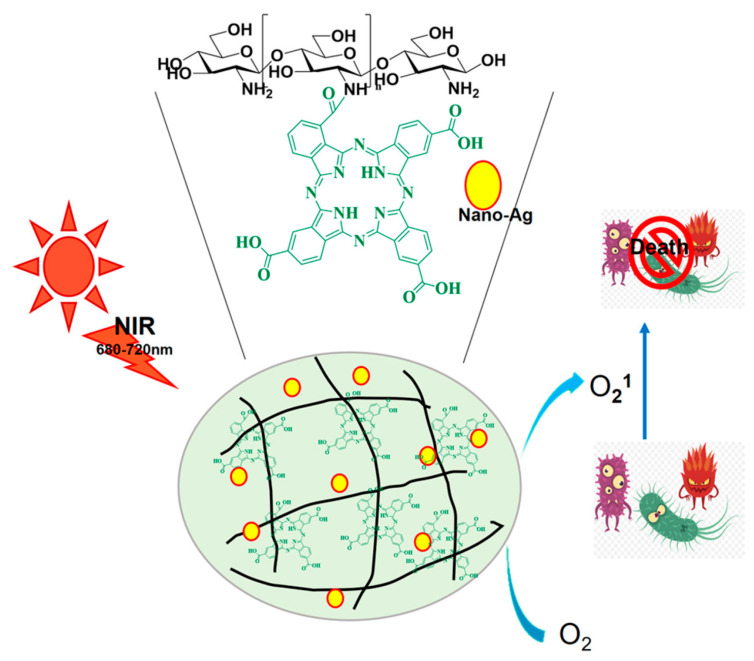
Mechanism of Pc-CS@Ag as photodynamic antibacterial agents against both Gram-positive and Gram-negative bacteria. Reproduced with authorization from the reference [24].

Using phthalocyanine-modified chitosan (Pc-CS) and nano-silver-doped Pc-CS@Ag as photodynamic antibacterial agents against both Gram-positive and Gram-negative bacteria, as shown in Figure 9, including drug-resistant strains, Wenqing Lai and colleagues produced and assessed. With EC90 values of 3.12–6.25 µg/mL, Pc-CS and Pc-CS@Ag efficiently inactivated bacteria, showing broad-spectrum antibacterial action [24]. In BALB/c mice, the materials clearly stimulated wound healing, showed concentration- and light intensity-dependent photodynamic action, and had a noticeable post-antibiotic effect (PAE). Confirmed was the formation of singlet oxygen and the destruction of bacterial cell walls induced by the ingredients, resulting in cell mortality. Compared to Pc-CS by itself, the nano-silver doping greatly raised antibacterial activity. Especially against drug-resistant bacteria, the research emphasizes the possibilities of these photosensitive materials as substitutes for antibiotics. The observation of singlet oxygen formation utilizing DPBF, the proof of bacterial death effectiveness, and the imaging of bacterial morphological alterations corroborate the research. The PAE results and in vivo wound healing data confirm the materials’ efficacy even more. Building on knowledge of photodynamic treatment mechanisms and silver nanoparticle antibacterial qualities, the study shows their combined benefit in this regard.

**Figure 10:**
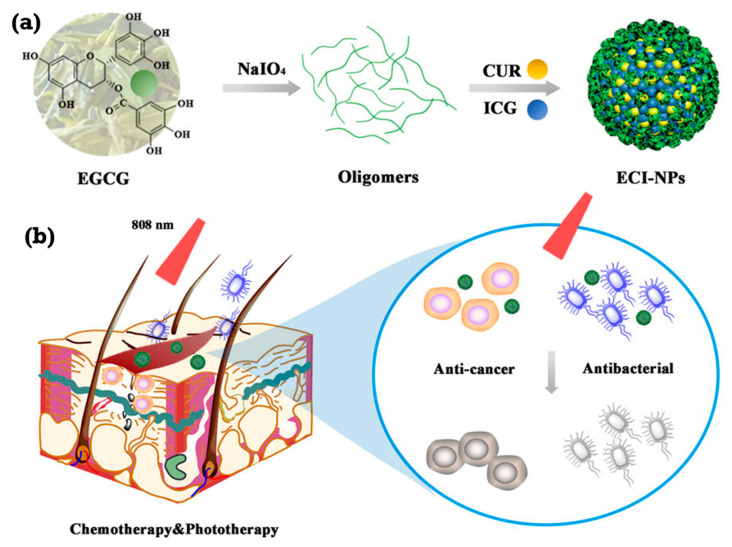
(**a**) EGCG oligomers, chemotherapeutic agent curcumin (CUR), and photosensitizer indocyanine green (ICG) generate a schematic representation of nanoparticles (ECI-NPs). (**b**) By means of near-infrared light-induced chemo-phototherapy, ECI-NPs show the therapeutic potential for postoperative infected wounds in tumor treatment by killing tumor cells and bacteria. Reproduced from an open-access reference [126].

Using targeting to create a dual-therapeutic platform for cancer and bacterial infections, Qi An and colleagues effectively synthesized multifunctional nanoparticles (ECI-NPs) constituted of Epigallocatechin gallate (EGCG) oligomers, Curcumin (CUR), and Indocyanine Green (ICG) by oxidative coupling [126]. As shown in Figure 10a, combining these elements was supposed to provide synergistic effects that would improve antibacterial and anticancer activities above those of separate components. With increased cellular absorption and phototoxicity in melanoma cells compared to free medicines, ECI-NPs show ideal stability, high drug loading, and regulated release. As shown in Figure 10b, their great antibacterial action demonstrated against *E. coli* and *S. aureus* likewise exceeded those of individual components. ECI-NPs improved the photostability and photodynamic/photothermal performance of ICG, elevated cellular uptake, and showed better anticancer effects and antibacterial activity. SEM, DLS, and UV-Vis spectroscopy indicated the effective production of E-NPs and ECI-NPs. Enhanced cellular damage and bacterial inhibition result from the mechanism of action being the combined chemotherapeutic effects of CUR and EGCG, together with the photothermal and photodynamic effects of ICG. The study concludes that ECI-NPs have great promise as new therapeutic agents for combination cancer and bacterial treatment; hence, more in vivo research and clinical translation are justified.

Using a new cationic amino acid porphyrin-based photosensitizer, both alone and in conjunction with an antibiotic, Zhanjuan Zhao and colleagues examined the therapeutic efficacy of photodynamic antimicrobial chemotherapy (PACT) for the treatment of diabetic foot ulcers (DFUs) in rats [127]. The theory was that the combined therapy would show better wound healing than any treatment taken on its own. Compared to the model, antibiotic-only, and PACT-only groups, the PACT + antibiotic group showed notably enhanced wound healing, angiogenesis (increasing VEGF), tissue maturation (reduced inflammatory cells), and bone regeneration (improved cortical bone volume and trabecular thickness). Improved bone repair was shown by micro-CT imaging, revealing the most notable periosteal reaction in the combo group. Consistent with the noted rates of wound healing, bacterial cultures revealed the lowest bacterial load in the combination group. The study concluded that PACT’s sensitizing effect on antibiotic treatment and increased antibiotic penetration from PACT-induced angiogenesis help to synergistically accelerate DFU healing. The data is backed by the noted changes in wound closure, bone characteristics, inflammatory markers, bacterial load, and tissue histology. Major organs were significantly unaffected by in vivo toxicity tests. The study supports earlier results on the function of VEGF in diabetic wound healing and the combined action of PACT with antibiotics, and justifies the use of the STZ-induced diabetic rat model.

**Figure 11:**
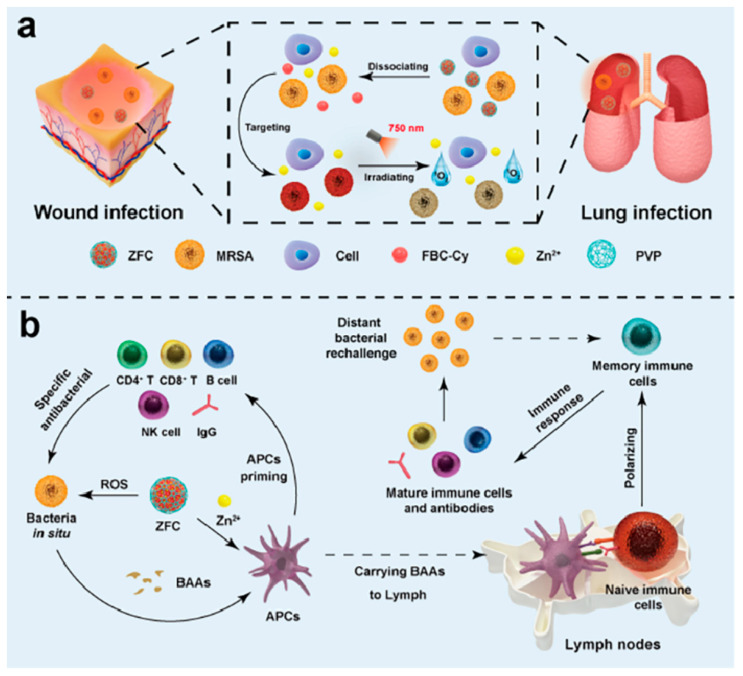
Mechanism of antimicrobial photodynamic-immune therapy using ZFC. (**a**) Synthesis and bactericidal mechanism of ZFC. ZFC is generated by coordinating Zn^2+^ with FBC-Cy, enabling metabolism-driven bacterial targeting and precise photobactericidal activity under 750 nm irradiation. (**b**) Activation of a bacteria-specific immune response by ZFC in a pulmonary bacterial infection model. Reproduced with authorization from the reference [128].

Designed for antimicrobial photodynamic-immune therapy (aPIT), Jiahao Zheng and colleagues present ZFC, a new near-infrared antimicrobial nanoplatform, utilizing bacterial metabolism to fight systemic bacterial infections [128]. The theory was that ZFC may generate particular bacterial targeting and eradication by using d-amino acid incorporation into bacterial peptidoglycans, therefore boosting a strong immune response at the same time. Under 750 nm light, the study showed that ZFC, which consists of d-cysteine-functionalized bacteriochlorin (FBC-Cy) and Zn^2+^, efficiently targeted and destroyed bacteria in wound and lung infections with minimal damage to normal cells, attaining over 90% bacterial clearance. By 3.2-fold, triggered systemic immunological responses and 1.84-fold, antibody expression increased, ZFC improved antigen-presenting cell activation, thereby creating immune memory. The mechanism, as shown in Figure 11, is ZFC dissociation via bacterial metabolism, releasing FBC-Cy for targeted ROS production and Zn^2+^ for further bacterial clearance. Targeting selected bacteria via d-amino acid incorporation, ZFC produced singlet oxygen and displayed improved antibacterial activity. Along with great biocompatibility, selective bacterial death, and strong immunological activation, ZFC has also been shown. These findings combine efficient bacterial clearance with increased immune activation to support the theory that ZFC can be a broad-spectrum treatment for systemic bacterial infections.

**Figure 12:**
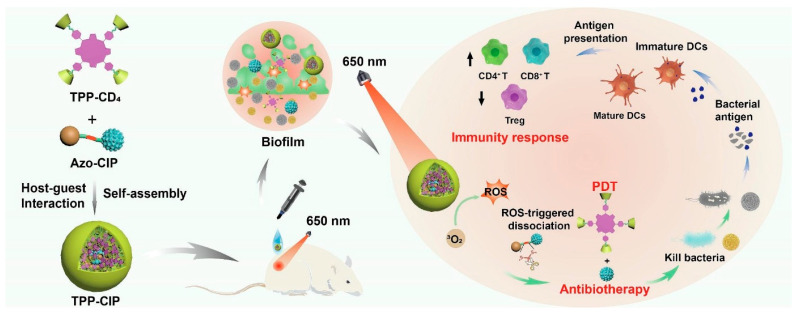
Mechanism of TPP-CIP NPs to accelerate wound healing in MRSA-infected mice [129].

To fight antibiotic resistance and biofilm development by photodynamic-immunotherapy and improved antibiotic penetration, Suwen Chen and colleagues created TPP-CIP NPs, porphyrin/ciprofloxacin-based supramolecular photosensitizer nanoparticles [129]. The theory was that light-activated ROS formation would cut thioketal linkers, releasing ciprofloxacin and inducing immunological responses, hence improving antibacterial activity. Formed via host–guest interactions, the NPs showed ROS-responsive antibiotic release, strong antibacterial efficacy against Gram-positive and Gram-negative bacteria, including drug-resistant strains, and efficient biofilm dispersal. Reduced inflammation and higher collagen deposition helped in vivo investigations of accelerated wound healing in MRSA-infected mice. As represented in Figure 12, the NPs also showed outstanding biocompatibility, improved DC and T cell activation, and M2 macrophage polarization. The mechanism consists of ROS-mediated bacterial disturbance, antibiotic release, and immunological stimulation, producing synergistic antibacterial actions and wound healing. Combining knowledge of PDT, antibiotic treatment, and immunotherapy, the research advances understanding of each and creates a single nanoplatform for better therapeutic results.

**Figure 13:**
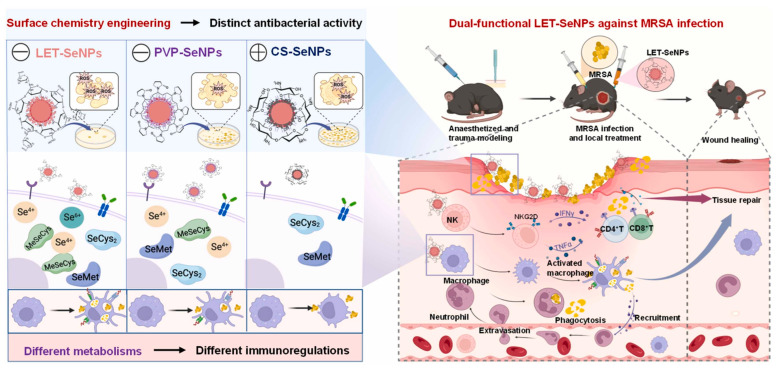
The schematic illustration of Se nanoparticles (SeNPs) with engineered surface chemistry as both bactericidal and immunoregulating agents against drug-resistant bacterial infection. Reproduced with authorization from the reference [130].

Qingyue Bu and colleagues hypothesized that by modifying the surface chemistry of selenium nanoparticles (SeNPs), a nanomaterial with dual bactericidal and immunomodulating properties might be produced, hence treating drug-resistant bacterial diseases [130]. By inducing strong ROS generation and damaging bacterial cell walls, the precisely engineered SeNPs using neutral (PVP), anionic (LET), and cationic (CS) surfactants showed the best inhibitory efficacy against methicillin-resistant *S. aureus* (MRSA). LET-SeNPs also greatly improved macrophage phagocytic capacity and efficiently triggered natural killer (NK) cells. By inducing higher infiltration of NK cells, CD8^+^ and CD4^+^ T lymphocytes into the infected area, in vivo experiments verified that LET-SeNPs greatly prevented MRSA infection and stimulated wound healing. The mechanism of the different metabolism of LET-SeNPs is depicted in Figure 13, which increases accumulation and specialized metabolite production (SeCys2, MeSeCys) in immune cells and damaged tissue, thereby improving selenoprotein synthesis and immune functioning. The study presented proof for the successful synthesis and characterization of functionalized SeNPs, exhibited better antibacterial activity of LET-SeNPs, showed increased immune cell activation, and confirmed in vivo therapeutic efficacy. This discovery supports the theory that LET-SeNPs can be a useful antibacterial agent with immunomodulating effects, therefore directing the creation of next-generation antibacterial treatments.

## 5. Recent Patents in Antibacterial Photodynamic Therapy

Besides academic advancement, the translational potential of antibacterial photodynamic therapy (aPDT) can also be approximated from recent patent reports. Several new patents disclose innovative materials and methods designed to enhance the efficacy, selectivity, and clinical applicability of PDT-based antimicrobial systems. For instance, as shown in Table 2, patent US-12121580-B2 discloses the use of copper–cysteamine nanoparticles for targeted aPDT with broad-spectrum antibacterial activity via enhanced ROS generation [131]. Similarly, US-2024366545-A1 from the University of Georgia [132] reports porphyrin-derived nanoparticles with improved photodynamic efficiency and biocompatibility. Non-invasive PDT device development is also evident in US-2023109074-A1 [133] and US-9526914-B2 [134], focusing on remote activation through up-conversion or infrared excitation to enable deeper tissue penetration. In parallel, US-10420346-B2 [135] demonstrates silver-nanoparticle-based photosensitizer enhancement through plasmonic effects, while EP-2198885-B1 [136] introduces calcium-phosphate nanoparticles as biocompatible dye carriers for PDT.

Other recent disclosures, such as US-2018055877-A1 [137], explore therapeutic nanoparticles with controlled release and targeting strategies. Earlier foundational patents like US20090326434A1 [18] and DE102014117532A1 [138] established the concept of antimicrobial PDT using sensitizer-delivery systems, whereas more recent filings (e.g., US20220409729A1 [139]) reveal optimized compositions with broad-spectrum microbial inactivation. Together, these patents reflect the dynamic innovation landscape of aPDT, where nanotechnology, photochemistry, and drug-delivery science are synergistically integrated to overcome traditional limitations in antimicrobial therapy.

In summary, the dominant trend across recent patents underscores a nanotechnology-driven approach that enhances ROS generation, enables deeper light activation, and improves biocompatibility marking a clear progression toward more efficient and clinically translatable antibacterial photodynamic systems.

## 6. In Vivo Applications of Different Nanoparticles in the Photodynamic Therapy

Photodynamic therapy (PDT) has also been an efficient, non-antibiotic method for battling bacterial infections in the context of increasing multidrug resistance. Underlying this breakthrough is the design of nanoparticle-based delivery platforms that provide increased loading of the photosensitizers, cell targeting to certain locations, and light-triggered antibacterial effect through reactive oxygen species (ROS) and, in some cases, synergizing agents such as nitric oxide (NO) or carbon monoxide (CO). Table 3 provides a comprehensive summary of recent in vivo studies employing such nanoparticles, highlighting their design strategies, efficacy, and relevance across models of infection. Several upconversion nanoparticle (UCNP)-based platforms have shown exceptional in vivo antibacterial activity. Zhang et al. created a UCNP@SiO_2_ platform with loading of TPE-Ph-DCM that could enable dual-action antibacterial treatment through ROS generation and NO release to completely kill *S. aureus* and *P. aeruginosa* in a rat keratitis model [140]. Similarly, Sun et al. employed UCNPs doped with a porphyrinic MOF (PCN-224) encapsulated in PVDF nanofibers to achieve over 99% bacterial reduction in a subcutaneous murine model [141]. Li et al. enhanced targeting by integrating lysozyme into mesoporous silica-coated UCNPs and achieving a 5.2-log reduction in MRSA viability [142]. Zhou et al. further explored this with PSeV-coated UCNPs, combining photothermal and photodynamic effects for 98% inhibition of MRSA in an inflammation-induced model [143]. Liu et al. introduced a CO-releasing nanoplatform (UCM@Si), loading Ce6 and Mn(CO)_5_Br, for antibacterial and anti-inflammatory properties with 80% inhibition of bacteria [37]. These illustrate the importance of introducing stimuli-responsive agents and multifunctionality into PDT platforms for useful in vivo antibacterial therapies.

Besides UCNPs, other systems have also demonstrated excellent therapeutic potential. Chu et al. prepared Cu-RCDs-C35 particles with carbon dots and quaternary ammonium compounds (QACs), which showed over 99% inhibition of bacteria and 96% wound healing in *S. aureus*-infected mice via a triple-synergistic PDT/PTT/QAC mechanism [20]. Qi et al. employed NO-releasing gold nanorods (GNRs@mSiO_2_-SNO) to induce a 4-log reduction in biofilms of *P. gingivalis* and *S. aureus* in a rat model of periodontal [144]. Wang et al. treated polymicrobial infections in peri-implantitis via a Ce6@ZIF-8-PDA/UBI nanoplatform, showing a ~73% inhibition vs. P. *gingivalis* and *F. nucleatum* [145]. Though a few nanostructures, such as UCNP@erythrosine [36], CS/N-CDs [19], curcumin hybrids [146,147], and copper sulfide-functionalized CDs [20] have not been attempted in vivo, the in vitro activity is extremely promising, having their bacterial inhibition of 91–100%. These results collectively demonstrate the therapeutic potential and flexibility of rationally engineered nanoparticles in antibacterial PDT, which justifies their further development towards clinical applications, as shown in Table 4.

## 7. Conclusions and Future Perspectives

In conclusion, the study of nanomaterial-mediated antibacterial photodynamic treatment (aPDT) is fast changing and has great potential to solve the expanding issues of bacterial infections, especially those resistant to traditional antibiotics. The reviewed research demonstrates that the integration of nanotechnology offers substantial, quantifiable advantages over conventional PDT using free photosensitizer molecules. The versatility of nanostructured materials, including carbon dots, mesoporous silica nanoparticles, liposomes, and upconverting nanoparticles, is crucial for overcoming the inherent limitations of free photosensitizers such as poor solubility, low stability, and lack of targeting. These nanoparticle systems have shown amazing capacity to increase photosensitizer administration, boost reactive oxygen species formation, and accomplish focused bacterial eradication. By combining aPDT with other therapeutic modalities such as photothermal therapy, chemodynamic therapy, and immunotherapy, the therapeutic potential has been further enhanced, resulting in synergistic effects and better treatment outcomes. Especially, these developments have aimed not only at bacterial eradication but also at wound healing promotion, inflammation reduction, and immune response stimulation, thus providing complete treatments for several medical disorders. The combined results highlight the transforming power of nanomaterial-mediated aPDT in transforming antimicrobial treatments, hence offering safer and more efficient substitutes for conventional antibiotics. Looking ahead, clinical translation and additional development in the realm of nanomaterial-mediated antibacterial photodynamic treatment have great promise. Future studies should concentrate on enhancing the design and synthesis of nanoplatforms to reach even greater accuracy in targeted distribution and controlled release of photosensitizers. Development of increasingly complex and successful therapeutic techniques depends on a better knowledge of the interactions of nanoparticles, bacteria, and the immune system. Promising directions are also the investigation of new photosensitizers with better photostability and biocompatibility. A critical consideration for the clinical translation of aPDT is the excitation wavelength. The studies reviewed employ a broad range, from short-wavelength blue/UV light (400 nm–500 nm) to long-wavelength red/NIR light (>650 nm) used by UCNPs and some liposomal systems. Whereas shorter wavelengths often generate ROS efficiently, they suffer from low tissue penetration and can induce undesired effects such as superficial photodamage or phototoxicity to host tissues. The use of NIR light offers the distinct benefit of deep-tissue activation and reduced host-tissue absorption, which is essential for treating deep-seated or systemic infections. Future work must prioritize NIR systems to achieve safe, deeply penetrating clinical applications and overcome these translational limitations. As well as the integration of artificial intelligence and machine learning for tailored aPDT, which could be leveraged for predicting optimal light dosage, photosensitizer concentration, and nanoparticle design based on patient-specific infection parameters and outcomes. Moreover, thorough preclinical and clinical studies are essential to confirm in practical environments the safety and efficiency of these nanoplatforms. Ensuring repeatability and enabling regulatory approval depends on the development of consistent procedures for nanomaterial synthesis, characterization, and evaluation. Furthermore, ensuring patient safety depends on the research of long-term consequences and possible toxicity of nanoparticles. Widespread acceptance of these novel treatments depends lastly on the extension of aPDT applications to solve other infectious diseases, including fungal and viral infections, and the development of scalable and reasonably priced production technologies. In the end, the ongoing development of nanomaterial-mediated aPDT will open the path for a new age of antimicrobial treatment, providing hope for addressing the worldwide challenge of antibiotic resistance and improving patient outcomes.

## Figures and Tables

**Table 1:** Illustrating major ROS mechanisms in the PDT antibacterial therapy.

Type	ROS	Generation Mechanisms	Antibacterial Mechanisms
Type II	^1^O_2_	Generated via energy transfer from excited photosensitizers or enzymatic oxidation (e.g., flavin-containing oxidases).	Induces oxidative damage to nucleic acids (purine oxidation), lipids (peroxidation of unsaturated fatty acids), and proteins (oxidation of amino acid residues).
Type I	OH·	Produced via Fenton/Haber-Weiss reactions, water radiolysis, or photocatalytic oxidation.	Highly reactive, causing DNA strand breaks, lipid peroxidation, and protein inactivation via amino acid oxidation.
	O_2_^−^	Formed by single-electron reduction of O_2_ via NADPH oxidase, mitochondrial respiration, or redox cycling.	Oxidizes thiol groups, disrupts Fe-S cluster enzymes, and propagates lipid peroxidation.
	H_2_O_2_	Generated via superoxide dismutation, oxidase-catalyzed reactions, or metabolic oxidative bursts.	Permeates bacterial membranes, oxidizes proteins, disrupts redox homeostasis, and generates ·OH via Fenton reactions.

**Table 2:** Illustrating the major patents filled and granted in last ten years PDT antibacterial therapy.

Patent Number	Title	Assignee(s)	Inventor(s)	Publication Date	Ref.
US-12121580-B2	Antibacterial photodynamic therapy using copper-cysteamine nanoparticles	Board Of Regents, the University of Texas System	Wei Chen	22 October 2024	[131]
US-2024366545-A1	Antibacterial porphyrin nanoparticles and methods for making and using the same	University of Georgia Research Foundation, Inc.	Hitesh Handa, Anil Kumar, Elizabeth J. Brisbois	7 November 2024	[132]
US-2023109074-A1	Non-invasive systems and methods for selective activation of photoreactive responses	Immunolight, Llc	Frederic A. Bourke, Jr., Zakaryae Fathi, Harold Walder	6 April 2023	[133]
US-9526914-B2	Non-invasive energy upconversion methods and systems	Duke University, Immunolight, Llc	Tuan Vo-Dinh, Jonathan P. SCAFFIDI, Venkata Gopal Reddy	27 December 2016	[134]
US-10420346-B2	Silver nanoparticle-enhanced photosensitizers	University of Cincinnati	Peng Zhang, Niranga Wijesiri, Hong Tang	24 September 2019	[135]
EP-2198885-B1	Calcium phosphate nanoparticles as dye carrier for photodynamic therapy	Biolitec AG, Universität Duisburg-Essen	Volker Dr. Prof. Albrecht, Burkhard Dr. Gitter, Susanna	8 February 2012	[136]
US-2018055877-A1	Therapeutic nanoparticles and methods thereof	Albert Einstein College of Medicine, Inc.	Joel M. Friedman, Mahantesh S. Navati, Adam J. Friedman, Parimala Nacharaju	1 March 2018	[137]
US20090326434A1	Anti-Microbial Photodynamic Therapy	n/a	Nikolay E. Nifantiev, et al.	22 December 2015	[18]
DE102014117532A1	Antimicrobial Photodynamic Therapy	n/a	Eva-Maria Decker, et al.	2 June 2016	[138]
US20220409729A1	Composition for Antimicrobial Photodynamic Therapy	n/a	Jimmie Kert	29 December 2022	[139]

**Table 3:** Illustrating the major characteristics of different nanoparticles used in PDT antibacterial therapy.

Nanoparticle	Advantages	Limitations	Applications in Photodynamic Antibacterial Therapy
Upconverting Nanoparticles (UCNPs)	NIR excitation for deep tissue penetration. Reduced photodamage to healthy tissue.	Low photothermal efficiency. Complex synthesis. Potential toxicity from rare-earth elements.	Used for deep tissue PDT, enhancing bacterial eradication in hard-to-reach infections via NIR excitation.
Carbon Dots (CDs)	High biocompatibility and low toxicity. Superior fluorescence and functionalization.	Moderate photodynamic efficiency. Stability issues. Scaling challenges.	Applied in antibacterial PDT, often functionalized for targeted therapy against specific bacterial strains.
Mesoporous Silica Nanoparticles (MSNs)	High surface area for drug loading. Biocompatible and biodegradable.	Limited photodynamic activity. Slow biodegradation.	Used as carriers for photosensitizers in PDT, enhancing drug delivery and multi-functional antibacterial therapy.
Polymer-Based Nanoparticles	Biodegradable, flexible design. High drug loading capacity.	Stability concerns. Costly synthesis.	Common in drug delivery systems for PDT, enabling targeted antibacterial treatment.
Liposomes	Biocompatible and biodegradable. Can encapsulate both hydrophilic and hydrophobic agents.	Expensive production. Premature leakage of encapsulated agents.	Used to encapsulate photosensitizers for efficient bacterial targeting and controlled release in PDT.

**Table 4:** In vivo applications of PDT based nanoparticles as antibacterial agents.

	Nanoparticle	Size (nm)	Photosensitizer	Synthesis Method	Design Strategy	In Vivo Model	Target Microorganism(s)	Laser Strength	Inhibition Values (%)	Novelty	Ref.
1	UCNP@SiO_2_	Hexagonal-phased (64.04 ± 1.81 nm)	TPE-Ph-DCM	Thermal decomposition	NaYF4:Yb3+, Tm3+@NaYF4:Nd3+, Yb3 doped with Mesoporous Silica.	SD rat cornea (Refractory keratitis)	*S. aureus* *P. aeruginosa*	808 nm at 0.4 W/cm^−2^ for 30 min	99% for both *P. aeruginosa* and *S. aureus*	Performed bacterial eradication with dual action of nitric oxide release and anti-inflammation.	[140]
2	UCNP@PCN@LA-PVDF	Hierarchical structure (130 nm)	Not Available	Electrospinning	NaYF_4_:Yb,Er nanorods doped with PCN-224 (MOF) in polyvinylidene fluoride.	Subcutaneous BALB/c mice model	*P. aeruginosa S. aureus*	980 nm at 0.5 W/cm^−2^ for 5 min	99.64% and 99.63% for *P. aeruginosa* and *S. aureus*	Nitric Oxide-assisted PDT.	[141]
3	UCMB-LYZ-HP	Crystalline hexanal 239 nm	Methylene blue	Solid liquid-thermal decomposition and silica sol–gel reaction	β-NaYF 4:Yb,Er@NaYF4 was doped with mesoporous silica and Lyzosome.	Murine Model	*MRSA*	980 nm, 0.5 W.cm^−2^, 10 min	5.2 log_10_ reduction in MRSA viability	Utilized lysozyme as an advanced antibacterial agent.	[142]
4	UCNPs/PSeV	hexagonal-phase 67 nm	Poly(selenoviologen) (PSeV)	Thermal decomposition-based self-assembly	PSeV was synthesized and further self-assembled with core–shell NaYF4:Yb/Tm@NaYF4.	MRSA-based Inflammation mouse model.	*MRSA*	980 nm, 0.150 W/cm^−2^, 4 min	98% CFU reduction	Synergistic PDT/PTT, accounted for a single NIR treatment.	[143]
5	UCNPs-Ce6-Mn(CO)5Br@Silane (UCM@Si)	Hexagonal Phase 78 ± 2 nm	Chlorin e6 (Ce6)	Solvothermal synthesis	NaErF_4_:Tm^3+^@NaYF_4_:Yb3^+,^ was first synthesized, which was further doped with Ce6 and Silane.	Subcutaneous abscess mouse model	*S. aureus*, *E. coli*	980 nm, 1 W·cm^−2^, 10 min	80% inhibition of *E. coli* and *S. aureus*	Synergistic (CO) mediated inflammation regulation and ROS production.	[37]
6	UCNP@mSiO_2_	Monodisperse orthohexagonal	Erythrosine (UCSE)	Solvothermal synthesis	NaYF_4_:Yb,Er@NaYF_4_ was first synthesized, which was further doped with erythrosine.	No in vivo studies	*S. aureus* *E. coli*	808 nm, 1 W/cm^2^, for 10 min	97.41 and 97.69% inhibition of *E. coli* and *S. aureus*	Erythrosine aided the photosensitization and ROS production.	[36]
7	CS/N-CDs	(2.34 nm–5.88 nm), diameter (3.80 ± 0.73 nm).	Nitrogen-doped carbon dots (N-CDs)	Solvent casting method, compounding N-CDs with chitosan (CS)	N-CDs were synthesized by adding different concentrations of Nitrogen to chitosan films.	No in vivo studies	*E. coli* *S. aureus*	White light for 6 h.	91.2% and 99.9% for *E. coli* and *S. aureus*	Enhanced antibacterial activity under UV-A light exposure; potential application in food packaging.	[19]
8	Cu-RCDs-C_35_	4.2 nm, graphene-like structure	(N-Cu-N) complex in Cu-RCDs-C_35_	Acid amine coupling of carbon dots and betaine	RCDs were first synthesized, followed by the Carbodiimide crosslinking with CAB-35.	Female Balb/c based (*S. aureus*) wound healing model.	*E. coli* *S. aureus*	808 nm irradiation (2.0 W/cm^2^, 5 min)	99.36% and 99.98%, for *E. coli* and *S. aureus*. 96% wound healing ratio	Triple synergistic sterilization combining QACs, PTT, and PDT under a single NIR light source	[20]
9	CDs/Cur	3 nm, spherical and uniform distribution	N, S-doped Carbon dots with curcumin	Hydrothermal route followed by dialysis-based purification	N, S-doped Carbon dots were first synthesized by the hydrothermal route, followed by the attachment of curcumin.	No in vivo studies	*E. coli* *S. aureus*	808 nm; 500 mW/cm^2^, 30 min	100% inhibition of *E. coli* for 1 μM CD/Cur	Dual PDT and PTT irradiation with fluorescent tracking and ROS effects.	[147]
10	CDs@Dop-CuS	Spherical, carbon dots of average particle size (6 nm)	Methylene blue	Microwave-assisted green synthesis, followed by solvothermal synthesis	Microwave irradiation facilitates rapid co-precipitation, yielding dopamine-functionalized copper sulfide nanoparticles, offering enhanced surface properties for potential applications.	No in vivo studies	*E. coli* *S. aureus*	laser diode (660 nm) and 808 nm 1.0 W/cm^2^	Enhanced singlet oxygen production, i.e., 79%	Synergistic photothermal and photodynamic effects; molecular docking-based theoretical insights provided.	[148]
11	GNRs@mSiO_2_-SNO	Cylindrical rods with an average size of 13.5 nm	Indocyanine green	Seed-mediated growth followed surface functionalisation and nitrosation.	Gold nanorods were synthesized, silica-coated for stability, functionalized with thiols, nitrosated for nitric oxide release, and drug-loaded for therapy.	Wistar rats based Periodontal inflammation model	*P. gingivalis* *S. aureus*	808 nm, 1 W cm^−2^, 5 min	4-log CFU reduction in biofilms	Triple-functional NO nanogenerator enabling synergistic aPDT/PTT/gas therapy for biofilm eradication.	[144]
12	UCNPs@mSiO_2_ (ZnPc)	High-temperature coprecipitation method	zinc phthalocyanine	Solvothermal and Stöber methods	Core–shell UCNPs@mSiO_2_ were synthesized via solvothermal and Stöber methods, then photosensitizer-loaded through adsorption.	No in vivo studies	*S. aureus* *E. coli*	980 nm, 1 W cm^−2^, 5 min	*S. aureus* and *E. coli* survival rates were 5.8% and 10.1%, respectively.	Combines chitosan for antibacterial, photodynamic wound healing therapy.	[149]
13	Ce6@ZIF-8-PDA/UBI	polyhedral shaped (99.9 ± 18.8 nm)	Chlorin e6 (Ce6)	Solvothermal method	Chlorin e6 (Ce6) was first absorbed on the surface of the ZIF structure, leading to the attachment of the PDA and UBI.	Rat femur peri-implantitis model [male SD rats (8 weeks old)]	*S. aureus* *E. coli* *F. nucleatum P. gingivalis*	660 nm, 5 min, 1.3 W/cm^2^	Significant decrese in 71.1% ± 0.9% (F. nucleatum) and 73.0% ± 2.0% (*P. gingivalis*) while	Synergistic photothermal and photodynamic effects with advanced photosensitizers like chlorin e6.	[145]
14	PDA-Cur (polydopamine-curcumin)	cluster-like structures (376 nm)	Curcumin (Cur)	Oxidative polymerization of dopamine followed by the adsorption of Curcumin	Cur adsorbed onto PDA through π-π stacking interactions and hydrogen bonding to form stable PDA-Cur complexes.	No in vivo studies	*E. coli* *S. aureus*	405 nm, 10 s, 0.5 mW/cm^2^	100% (*E. coli* and *S. aureus*)	Dual PDT and PTT irradiation with Cur-based fluorescent tracking and ROS effects.	[146]

(The logarithmic reduction in viable cells (log CFU reduction) can be converted to percentage inhibition using the following relationship: $\mathrm{Percentage}\;\mathrm{Inhibition}=\left(1-\frac{1}{10^{\log\;\mathrm{reduction}}}\right)\times 100$. For example, a 4-log CFU reduction corresponds to a 99.99% inhibition of biofilm growth, indicating a 10,000-fold decrease in viable cells).

## Data Availability

No new data were created or analyzed in this study. Data sharing does not apply to this article.
